# Percutaneous endoscopic drainage for acute long segment epidural abscess following endoscopic lumbar discectomy: A case report

**DOI:** 10.3389/fsurg.2022.985666

**Published:** 2022-09-30

**Authors:** Tao Li, Hui Wu, Jinghong Yuan, Jingyu Jia, Tianlong Wu, Xigao Cheng

**Affiliations:** ^1^ Department of Orthopedics, The Second Affiliated Hospital of Nanchang University, Nanchang, China; ^2^ Institute of Orthopedics of Jiangxi Province, Nanchang, China; ^3^ Institute of Minimally Invasive Orthopedics of Nanchang University, Nanchang, China

**Keywords:** epidural abscess, percutaneous endoscopic drainage, PELD = percutaneous endoscopic lumbar discectomy, case report, minimal invasive

## Abstract

**Introduction:**

Acute epidural abscess after percutaneous endoscopic lumbar discectomy is a rare but grievous complication. When faced with a long-segment epidural abscess, open surgery has traditionally been performed which can lead to huge surgical trauma and unpredictable complications. For this reason, surgeons around the world are constantly looking for more minimally invasive and effective surgical methods.

**Patient Concerns:**

Our patient was a 32-year-old woman who had been receiving percutaneous endoscopic interlaminar discectomy for L5/S1 lumbar disc herniation one week ago. She returned to our institution with a fever and lower back pain.

**Diagnoses:**

Magnetic resonance imaging revealed a long segment epidural abscess accompanied by a paravertebral abscess, and staphylococcus aureus was detected in a bacterial culture of pyogenic fluids extracted from the paravertebral abscess.

**Treatments:**

We performed percutaneous endoscopic drainage (PED) for the epidural abscess. Long-term sensitive antibiotic treatment after surgery.

**Outcomes:**

Immediate pain relief was achieved and the inflammatory reaction subsided after 4 weeks of antibiotic therapy. Re-examination of the lumbar spine MRI after 1 month showed that the epidural abscess disappeared completely.

**Conclusion:**

Percutaneous endoscopy allowed us to approach the epidural abscess directly, enabling the immediate drainage of the abscess with minimal trauma to the patient. The good results obtained show that percutaneous endoscopic drainage is a reliable way to treat a long-segment epidural abscess.

## Introduction

Epidural abscess is a rare and severe complication of percutaneous endoscopic lumbar discectomy (PELD) (1) that can result in serious vascular and nerve injury if not detected and treated promptly (2–4). Antibiotic therapy alone is the first choice when confronted with this situation. Nevertheless, drainage is recommended for cases involving large abscesses or when antibiotic therapy is ineffective and open surgery has traditionally been performed (5, 6). Hereby We present a case of acute long segment epidural abscess following PELD that was successfully treated by percutaneous endoscopic drainage.

## Case presentation

### Chief complaints

A 32 years old young Asia woman initially presented with low back pain, left lower limb numbness and fever.

### History of present illness

The patient had a fever for one week. Four days ago, the patient developed low back pain accompanied by numbness and severe pain in the left lower limb.

### History of past illness

The patient had transforaminal endoscopic treatment for disc herniation one week ago (day surgery). There was no additional medical, family or genetic history.

### Physical examination

Physical examination on admission, the patient's temperature was 38.1 °C, heart rate was 88 bpm, respiratory rate was 20 breaths per minute, blood pressure was 110/83 mmHg. The patient has significant tenderness in the lower back. The muscle strength of the left lower limb was grade III.

### Laboratory examinations

Blood tests on admission: white blood cell count 19.01 × 10^9^, hemoglobin 97 g/L, percentage of neutrophils 90%, red blood cell count and platelet count was normal. C-reactive protein greater than 200 mg/L, ESR was 78 mm/h. Albumin was 29.58 g/L, and the albumin ratio was 1.04. The electrocardiogram and chest X-ray results were normal.

### Imaging examinations

**Figure 1:**
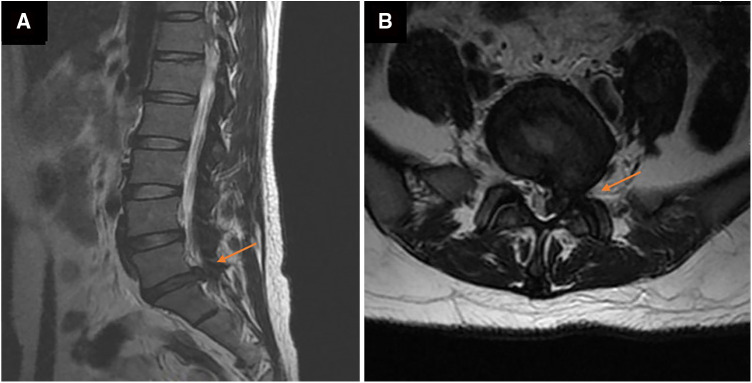
Magnetic resonance images of the lumbar before percutaneous endoscopic lumbar discectomy (PELD) in axial (**A**) and sagittal (**B**). a large disc herniation with compression of the spinal cord and nerve roots at the left of L5/S1.

**Figure 2:**
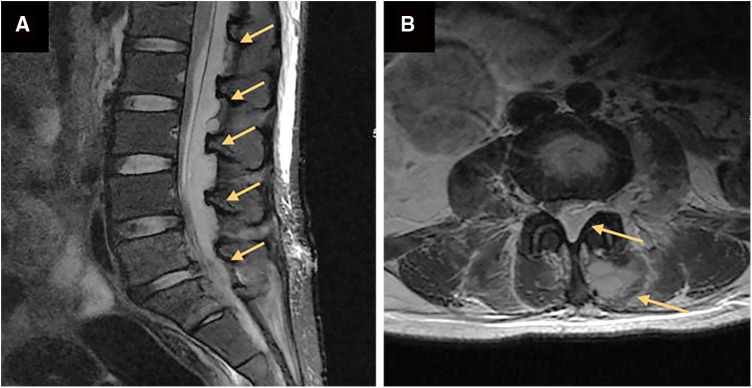
Magnetic resonance images of the lumbar before percutaneous endoscopic drainage in axial (**A**) and sagittal (**B**). (**A**) a long segment epidural abscess from L1 to S1 (orange arrow). (**B**) Massive epidural abscesses compressing the spinal cord and nerve roots with paravertebral involvement (orange arrow).

A herniated L5-S1 disc with compression of the spinal cord and the left nerve root was found by magnetic resonance imaging (MRI) of the lumbar spine a week ago (Figure 1). Magnetic resonance imaging on admission demonstrated long-segment hyperintensity in the epidural and paravertebral. The pus ranges from L1 to S1 in sagittal and the dura mater was compressed to about 1/3 of its original value in axial. (Figures 2A,B).

### Microbiological identification of the causative agent

X-ray guided aspiration of pus and bacterial culture and drug sensitivity test was performed. The bacterial culture revealed a Staphylococcus aureus infection, and the drug sensitivity test showed that MOXIFLOXACIN is effective.

## Final diagnosis

The final diagnosis of the presented case is Acute epidural abscess caused by Staphylococcus aureus infection after endoscopic discectomy.

## Treatment

**Figure 3:**
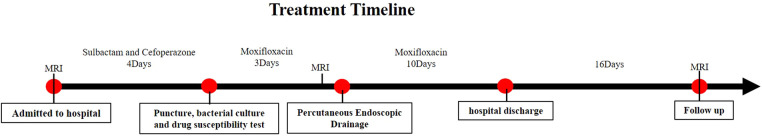
The treatment timeline of this visit.

Antibiotic treatment: The whole antibiotic process was shown in Figure 3. The C-reactive protein was >200 mg/L, the white blood cell count was 19.01 × 10^9^/L, the ESR was 78 mm/h, and the body temperature was 38.1 °C on admission. The patient was treated with cefoperazone sodium and sulbactam sodium. Moxifloxacin was replaced for antibacterial treatment four days later, depending on the results of bacterial culture and drug sensitivity test following a puncture. Three days later, the C-reactive protein was 173.9 mg/L, the white blood cell count was 27.71 × 10^9^/L, the ESR was 117 mm/h, the body temperature was 38.5 °C and percutaneous endoscopic drainage was performed immediately. The pus extracted during the operation was subjected to bacterial culture and drug susceptibility test, and the results were the same as before (Moxifloxacin is sensitive, MIC≤0.25). Moxifloxacin was continued until discharge.

**Figure 4:**
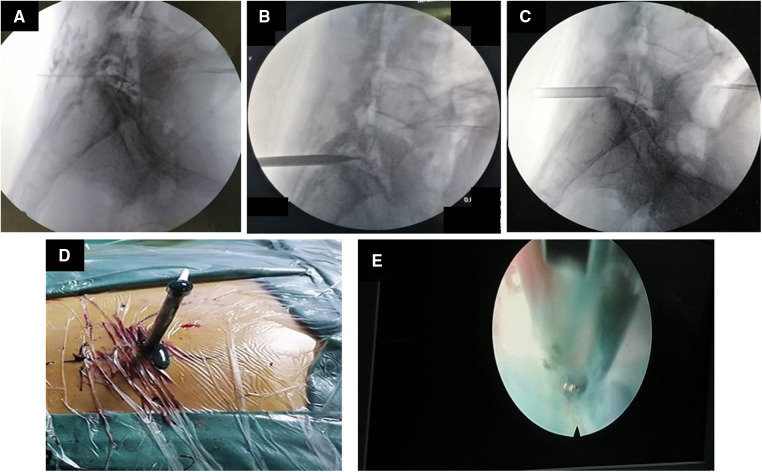
Images and video during percutaneous endoscopic drainage. (**A**) The guide wire is positioned at L5/S1. (**B**) Insert the channel along the guide wire. (**C**) The channel is placed at L5/S1. (**D**) After the passage is placed, pus begins to flow out. (**E**) Microscopic video of massive pus discharge.

**Figure 5:**
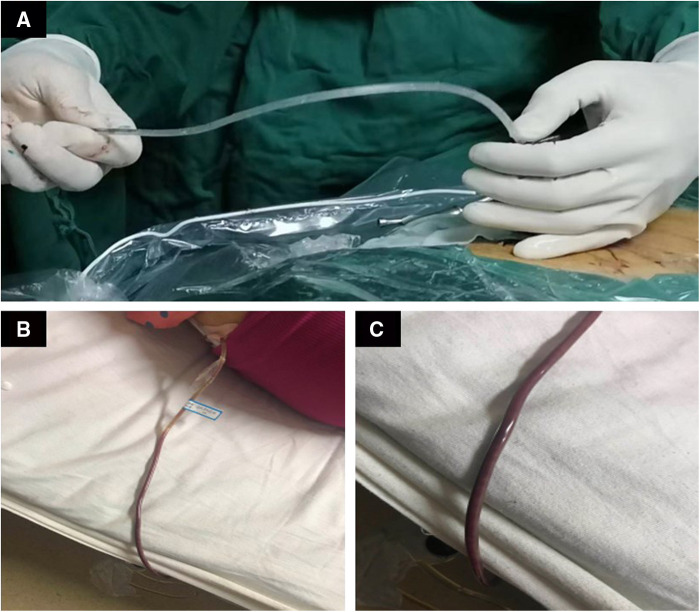
Postoperative drainage of pus.

Surgery treatment: Patients were prone position, head high and feet low. Dezocine was given intravenously during the operation. The operation area was disinfected, covered with sterile cloth and pasted with skin film. Before the procedure, lidocaine was used for local anesthesia, and the guide wire was placed at the left intervertebral foramen of L5/S1 by cutting the skin 0.6 cm at the root of the guide wire. Also, establish the working channel along the guide wire. At this point, a small amount of pus can be seen flowing out of the pipeline (Figures 4A–C). Then the light source and the camera lens were connected, put the endoscope into the cannula, and adjusted with a proper water flow and pressure. The necrotic fibrous tissue around the abscess was removed with nucleus pulposus forceps, and the intervertebral foramen were appropriately enlarged, and a large amount of pus was seen gushing out (Figures 4D,E and Supplementary 1). Irrigation and drainage with normal saline, until no obvious pus outflow, visible dura swelling, nerve root relaxation. Than Place a drainage tube along the working channel, check pipe patency, exit the working channel, fix the drainage tube, suture the wound, and cover it with sterile dressing (Figure 5A). Check the patency of the drainage tube daily (Figures 5B,C) and observe the patient closely.

### Outcome and follow-up

**Figure 6:**
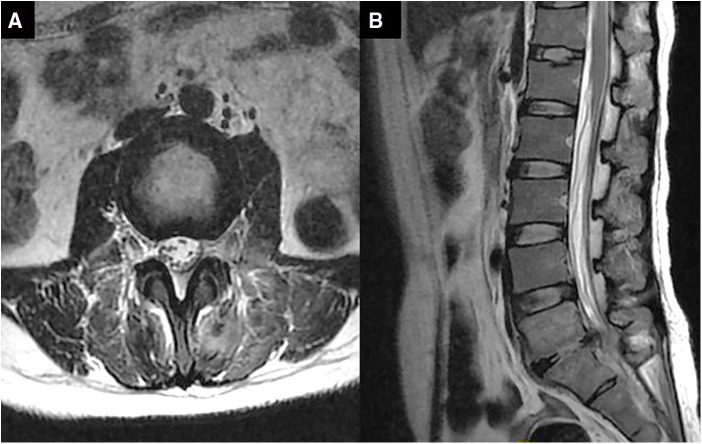
Magnetic resonance images of the lumbar vertebra after percutaneous endoscopic drainage for 1 month in axial (**A**) and sagittal (**B**). intraspinal and paravertebral abscesses have disappeared.

The patient's waist and left lower limb symptoms were significantly alleviated after the operation. One week later, the patient's body temperature dropped to normal (37.0 °C). About ten days later, the erythrocyte sedimentation rate (17 mm/h) and C-reactive protein(10 mg/L) decreased to normal. Two weeks later, the patient's symptoms disappeared, and she was discharged. Reexamination of lumbar magnetic resonance one month after surgery showed that the patient had complete disappearance of intraspinal and paravertebral abscesses (Figure 6).

## Discussion

Infection after PELD is a rare but serious complication, with an incidence of 0.1% to 0.7% (7), Such as Gu et al. treated 209 patients with lumbar disc herniation with minimally invasive surgery, and one patient developed a lumbar infection, with an infection rate of 0.47% (8). Fan et al. treated 738 patients with lumbar spinal stenosis with PELD, and 3 patients developed infection, with an infection rate of 0.41% (9). But Zhou et al. treated 426 patients with lumbar disc herniation with PELD and PEID without lumbar infection (1).

For lumbar epidural infection, the most important is active antimicrobial treatment and timely abscess removal. Antimicrobial therapy is the basis of treatment, and surgical decompression is always the key to the treatment of epidural abscess, especially when accompanied by neurological impairment (2, 10–13). David E Connor Jr et al. treated 77 patients with epidural abscesses surgically, with significant improvement in 56 patients and some improvement in symptoms in 6 patients (12). Amit R Patel et al., who treated 128 patients with epidural abscesses with medical therapy alone (51 patients) and surgery plus antibiotics (77 patients), showed that early surgery improved neurological outcomes, with more than 41% of patients requiring surgical decompression after medical therapy failed (14).

The treatment strategy for infected spine remains controversial. Conservative cases appear to be more likely to develop mechanical back pain and develop more deformities over time than surgical cases (15). However, open surgical intervention is always associated with more complications, despite lower overall mortality among surgical patients (16). It can cause huge surgical trauma, requiring the dissection of paravertebral muscles and the biting of lamina, seriously damaging the stability of the spine, and sometimes even requiring fusion surgery, which can reduce the patients' lumbar range of motion and greatly reduce the patients' quality of life (17). PED has the advantages of less complications and satisfactory clinical efficacy, which provides a minimally invasive surgical option for the treatment of spinal infection (18–20).

Percutaneous endoscopic drainage is an attempt of spinal endoscopy in the treatment of spinal infections. Choi et al. used percutaneous endoscopic drainage to treat four different types of spinal infections, and most patients achieved satisfactory results (14/17). Among them, 4 cases were used to treat epidural abscesses, 2 patients achieved satisfactory results, 1 patient underwent minimally invasive drainage here (21), and 1 patient underwent open surgery here. In addition, Akira Iwata et al. used PED to successfully treat 4 patients with spinal fungal infection (18). Kai-Sheng Chang and Omar S Akbik respectively drainage of the cervical and thoracic epidural abscess through transforaminal endoscopy and received excellent result (22, 23).

Endoscopic treatment of epidural abscess has certain limitations. Endoscopic debridement of epidural abscesses may be limited in scope compared to traditional open surgery and may require reoperation to allow complete removal of pus.The success of PED treatment largely depends on the removal of pus that causes spinal cord compression and nerve damage. Endoscopic treatment alone requires more than a certain level of endoscopic surgical skill, so treatment outcomes may vary depending on the skill of the operator.

In this case, the long-stage abscess compressed the spinal cord and nerves, and the patient had severe neurological symptoms, so immediate spinal decompression was necessary. We planned to perform PED drainage first. If the neurological symptoms did not improve after endoscopic treatment of the epidural abscess, additional posterior spinal open surgery could be considered at any time. Fortunately, the patient's symptoms improved significantly after the operation, and the validation index gradually decreased. At the last follow-up, the collapse of the L5/S1 intervertebral space was observed, but this patient did not have any local or global symptoms.

Nowadays, minimally invasive surgery is gradually replacing the conventional open surgery field, and then open surgery is needed in the field of epidural infection, minimally invasive surgery can also achieve better surgical results. This means that the area in which endoscopic technology can be used has been widened. For some skilled surgeons in endoscopic surgery, this technique may be one of the good treatment options.

## Conclusion

We could approach the epidural abscess directly with percutaneous endoscopy, allowing for immediate drainage of the abscess with less burden on the patient. The success of this case demonstrates that transforaminal endoscopy can be used to treat long segment spinal epidural abscess, which can be used as a reference for clinicians when developing a surgical plan for the treatment of acute long segment spinal epidural abscess.

## Care checklist (2013) statement

The authors have read the CARE Checklist (2013), and the manuscript was prepared and revised according to the CARE Checklist (2013).

## Data Availability

The raw data supporting the conclusions of this article will be made available by the authors, without undue reservation.
